# Clinical and molecular features of children with Beckwith-Wiedemann syndrome in China: a single-center retrospective cohort study

**DOI:** 10.1186/s13052-020-0819-3

**Published:** 2020-04-29

**Authors:** Ruixue Wang, Yongmei Xiao, Dan Li, Hui Hu, Xiaolu Li, Ting Ge, Ronghua Yu, Yizhong Wang, Ting Zhang

**Affiliations:** Department of Gastroenterology, Hepatology and Nutrition, Shanghai Children’s Hospital, Shanghai Jiao Tong University, Shanghai, 200062 China

**Keywords:** Beckwith-Wiedemann syndrome, Chromosome 11p15.5, Imprinting center, Macroglossia, Methylation abnormality

## Abstract

**Background:**

Beckwith-Wiedemann syndrome (BWS) is a genetic overgrowth disorder with variable clinical features and cancer predisposition. In this study, we aim to characterize the clinical features and molecular defects of BWS patients in China.

**Methods:**

Thirty-one patients with clinical suspicion of BWS were retrospectively recruited to the study from Shanghai Children’s Hospital between January 2014 and December 2017. Clinical data, including demographics, clinical features, and molecular testing results were extracted and systematically analyzed.

**Results:**

Twenty-one patients with a BWS score ≥ 4 (6, IQR 4, 7) were clinically diagnosed with BWS, and 10 children with a BWS score ≥ 2 and < 4 (2, IQR 2, 3) were clinically suspected BWS patients. The most common cardinal feature of clinically diagnosed patients was macroglossia (71.4%) followed by lateralized overgrowth (33.3%) and exomphalos (14.3%), and the major suggestive features were umbilical hernia and/or diastasis recti (65.0%) and ear creases or pits (61.9%). Among 10 clinically suspected BWS patients, macroglossia and lateralized overgrowth were observed in 3 (30%) and 2 (20%) patients, and umbilical hernia and/or diastasis recti occurred in 7 (70.0%) patients. Seven (33.3%) clinically diagnosed patients and 3 (30%) suspected patients were identified with loss of methylation at KCNQ1OT1:TSS differentially methylated region (DMR; IC2 LOM), 5 (23.8%) clinically diagnosed BWS patients were identified with gain of methylation at H19/IGF2:IG-DMR (IC1 GOM), and 1 (4.8%) clinically diagnosed BWS patients was identified with paternal uniparental isodisomy 11 (pUPD11). The phenotype-genotype correlation analysis showed no significant difference among patients with IC2 LOM, IC1 GOM, and pUPD11.

**Conclusions:**

The current study presents the first cohort study of BWS patients in mainland China. The clinical and molecular features of the patients are similar to those of other reported BWS patients in the Chinese population.

## Introduction

Beckwith-Wiedemann syndrome (BWS, OMIM#130650), first reported by Beckwith JB and Wiedemann HR in the 1960s, is a rare genetic overgrowth disorder with variable clinical features and cancer predisposition [1–3]. The estimated incidence of BWS is 1 in 10,000 live births [4]. The clinical manifestations of BWS include macroglossia, macrosomia, abdominal wall defects, hemihyperplasia, enlarged abdominal organs, ear anomalies, facial nevus flammeus, and nephroureteral malformations. In addition, an increased risk of developing embryonal tumors during early childhood was reported in patients with BWS, such as Wilms’ tumor and hepatoblastoma [1, 5].

BWS is caused mainly by molecular alterations affecting imprinted gene expression located within the chromosome 11p15.5 region [1, 2]. The imprinting cluster of chromosome 11p15.5 harbors two imprinting domains, IGF2/H19 and CDKN1C/KCNQ1/KCNQ1OT1, which are controlled by H19-associated imprinting center 1 (IC1) and KCNQ1OT1-associated IC2, respectively [6]. It has been demonstrated that an epigenetic or genetic defect affecting imprinted genes in chromosome region 11p15 could be observed in the majority of BWS patients, and DNA methylation abnormalities are the most commonly detected molecular defects [7, 8]. Gain of methylation at H19/IGF2:IG differentially methylated region (DMR; IC1 GOM), loss of methylation at KCNQ1OT1:TSS (DMR; IC2 LOM), paternal uniparental isodisomy (pUPD11), CDKN1C loss of function mutations, and chromosome abnormalities altering copy number or structure of 11p15.5 were identified in reported BWS patients [4, 7–9].

Various clinical phenotypes and genotypes have been well-described in European and North American BWS patients [1]. However, there are only limited reports of the clinical features and molecular etiology of BWS patients from mainland, China. In this study, we conduct a single-center retrospective study to characterize the clinical features and genetic defects of patients with clinical suspicion of BWS in Shanghai, China.

## Materials and methods

### Study cohort

Thirty-one children with clinical suspicion of BWS enrolled in Shanghai Children’s Hospital were retrospectively recruited to the study between January 2014 and December 2017. Clinical data including demographics, clinical features, pregnancy-related findings, family history of BWS, and molecular testing results were extracted from medical records and were systematically reevaluated by a recently developed international consensus statement of clinical and molecular diagnosis of BWS [1]. The clinical diagnostic criteria of classical BWS are patients with a BWS score of ≥4 based on cardinal and suggestive features. The scoring system is defined as 2 points per cardinal feature and 1 point per suggestive feature [1]. This study was conducted in compliance with the Helsinki Declaration and was approved by the Ethical Review Board of Shanghai Children’s Hospital. Written informed consent was obtained from parents or legal guardians of all pediatric patients.

### Molecular testing

Genomic DNA was extracted from peripheral blood of the subject using the QIAamp DNA Mini Kit (Qiagen, Hilden, Germany). The methylation-specific-multiplex ligation-dependent probe amplification (MS-MLPA) method was used to detect the methylation status and copy number change of the IC1 (H19/IGF2:IG-DMR) and IC2 (KCNQ1OT1:TSS-DMR) genes in the chromosome 11p15 region. The kit used was ME030-C1 BWS/RSS kit from MRC-Holland (Amsterdam, NL) in accordance with the manufacturer’s instructions. Genomic DNA (200 ng) was denatured and hybridized for 16 h at 60°Cby the probe mixture. Samples were equally split into two aliquots and treated either with ligase alone or with ligase and HhaI. Polymerase chain reaction (PCR) was performed and the products were separated on ABI Dx3500 genetic analyzer (Applied Biosystems, Foster City, CA, USA). Genescan software was used to analyze the electropherograms and Coffalyser version 9.4 software (MRC-Holland, Amsterdam, NL) was used to calculate the relative peak area. Heterozygous deletions or duplications of recognition sequences were defined as a 35–50% reduced relative peak height of the amplification product of that probe. GOM and LOM was defined as a methylation percentage > 20% higher or lower than the normal healthy control (methylation percentage around 50%), respectively [10]. Patient had both IC1 GOM and IC2 LOM is identified as pUPD11 [11].

### Data analysis

Demographics, clinical features, pregnancy-related findings, family history of BWS, and molecular testing results of the patients were collected for analysis. All data were entered into a customized database and then analyzed with SPSS statistical software (version 22, IBM, Armonk, NY, USA). Quantitative data were summarized as the median and interquartile range (IQR 25th–75th) or number with percentage where appropriate. Descriptive analysis was conducted to analyze the general characteristics, specific clinical features, and molecular defects of the patients. Comparison of clinical features among the different genotype groups was conducted by using Fisher’ s exact tests (2 × 2, or 3 × 2 matrices). Two-tailed *p*-values ≤0.05 were considered as statistically significant.

## Results

### Clinical features

**Fig. 1:**
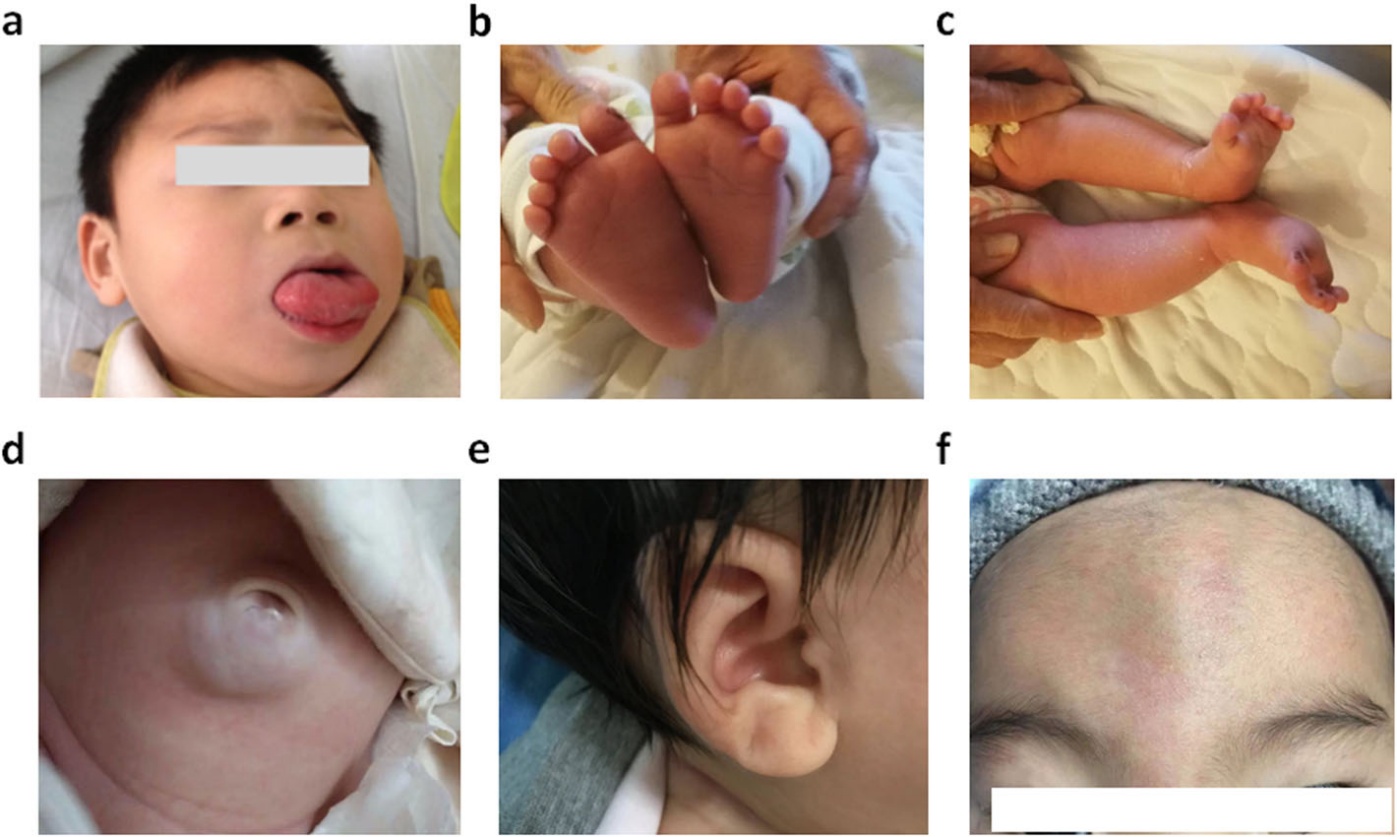
Representative cardinal and suggestive features of Beckwith-Wiedemann syndrome: **a** Macroglossia (patient No. 3); **b**, **c** Lateralized overgrowth (patient No. 7); **d** Umbilical hernia (patient No. 1); **e** Ear creases (patient No. 13); (**f**) Facial naevus simplex (patient No. 23). Written informed consent was obtained from the parents for the publication of these images

As shown in Table 1, of those 31 children with clinical suspicion of BWS enrolled in the study, 18 were boys (58.1%), 13 were girls (41.9%), and the median age at enrollment was 3 months (IQR 2, 6.5), ranging from 1 to 72 months. Twenty-one children with a BWS score ≥ 4 (6, IQR 4, 7) were clinically diagnosed with BWS, and 10 children with a BWS score ≥ 2 and < 4 (2, IQR 2, 3) were clinically suspected BWS patients. The most common cardinal feature of 21 clinically diagnosed patients was macroglossia (Fig. 1a), which was observed in 15 patients (71.4%) followed by lateralized overgrowth (7/21, 33.3%; Fig. 1b, c) and exomphalos (3/21, 14.3%). Hyperinsulinism lasting > 1 week and requiring escalated treatment was observed in 1 patient (4.7%). The most common suggestive features were umbilical hernia (Fig. 1d) and/or diastasis recti and ear creases or pits (Fig. 1e), which were detected in 65.0 and 61.9% of the clinically diagnosed patients, respectively. Other suggestive features were also observed in clinically diagnosed BWS patients: facial naevus simplex (11/21, 52.3%, Fig. 1f), nephromegaly and/or hepatomegaly (10/21, 47.6%), birthweight > 2 SDS above the mean (8/21, 38.1%), transient hypoglycemia (5/21, 23.8%), and polyhydramnios (4/21, 19.0%). Among 10 clinically suspected BWS patients, macroglossia and lateralized overgrowth were observed in 3 (30.0%) and 2 (20.0%) patients, respectively. The most common suggestive feature of clinically suspected BWS patients was umbilical hernia and/or diastasis recti (7/10, 70%) followed by nephromegaly and/or hepatomegaly (2/10, 20.0%), birthweight > 2 SDS above the mean (2/10, 20.0%), ear creases or pits (1/10, 10.0%), and polyhydramnios (1/10, 10.0%). BWS-related embryonal tumors were not observed at the time of enrollment in this cohort. One female patient with a BWS score of 6 was conceived by assisted reproductive technology (ART).

**Table 1:** Clinical features of patients with clinical diagnosis of BWS (*n* = 21, BWS score ≥ 4) or suspected BWS (*n* = 10, BWS score ≥ 2 and < 4)

	Clinical diagnosis	Suspected	Total
	*n* = 21	(*n* = 10)	(*n* = 31)
Age, months, median (IQR)	4 (2, 12)	2.5 (1, 4.5)	3 (2, 6.5)
Gender			
Boy	13 (61.9%)	5 (50.0%)	18 (58.1%)
Girl	8 (38.1%)	5 (50.0%)	13 (41.9%)
BWS score, median (IQR)	6 (4, 7)	2 (2, 3)	4 (3, 6)
Cardinal features			
Macroglossia	15 (71.4%)	3 (30%)	18 (58.1%)
Lateralized overgrowth	7 (33.3%)	2 (20%)	9 (29.0%)
Exomphalos	3 (14.3%)	0 (0%)	3 (9.7%)
Hyperinsulinism	1 (4.7%)	0 (0%)	1 (3.2%)
Wilms tumor or nephroblastomatosis^a^	0 (0%)	0 (0%)	0 (0%)
Suggestive features			
Umbilical hernia and/or diastasis recti	16 (65.0%)	7 (70.0%)	23 (74.2%)
Ear creases and/or pits	13 (61.9%)	1 (10.0%)	14 (45.2%)
Nephromegaly and/or hepatomegaly	10 (47.6%)	2 (20.0%)	12 (38.7%)
Facial naevus simplex	11 (52.3)	0 (0%)	11 (35.5%)
Birthweight > 2 SDS above the mean	8 (38.1%)	2 (20.0%)	10 (32.3%)
Transient hypoglycaemia	5 (23.8%)	0 (0%)	5 (16.1%)
Polyhydramnios	4 (19.0%)	1 (10.0%)	5 (16.1%)
Typical BWS tumors^b^	0 (0%)	0 (0%)	0 (0%)

^a^ Multifocal and/or bilateral Wilms tumor or nephroblastomatosis

^b^ Neuroblastoma, rhabdomyosarcoma, unilateral Wilms tumour, hepatoblastoma, adrenocortical carcinoma or phaeochromocytoma

*BWS* Beckwith-Wiedemann syndrome, *IQR* interquartile range

### Molecular defects

**Fig. 2:**
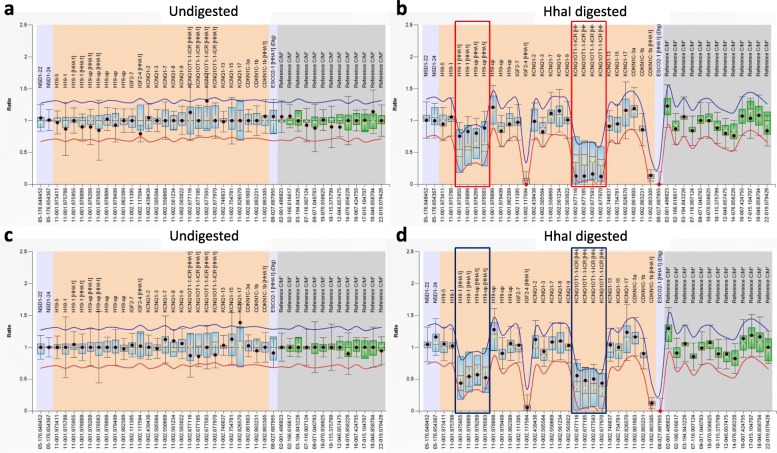
MS-MLPA test result of the patient (No. 19) had both gain of methylation at H19/IGF2:IG differentially methylated region (DMR; IC1 GOM) and loss of methylation at KCNQ1OT1:TSS-DMR (IC2 LOM) (**a**, **b**), and a healthy control (**c**, **d**)

The MS-MLPA test was performed in all 31 patients with clinical suspicion of BWS. A total of 10 (7 clinically diagnosed and 3 suspected) patients were identified with IC2 LOM, and 5 clinically diagnosed BWS patients were identified with IC1 GOM (Table 2). One 4-year old boy had both IC1 GOM and IC2 LOM, was identified as pUPD11 (Fig. 2). The female patient conceived by ART was identified with IC2 LOM. No copy number change of the IC1 and IC2 was observed by MS-MLPA. The correlation analysis between clinical features and genotype showed no significant difference among patients with IC2 LOM, IC1 GOM, and pUPD11, except lateralized overgrowth (Table 3). Finally, a total of 24 patients were confirmed with BWS based on a BWS score ≥ 4 and/or a detected molecular defect.

**Table 2:** Molecular defects of patients with clinical diagnosis of BWS (*n* = 21, BWS score ≥ 4) or suspected BWS (*n* = 10, BWS score ≥ 2 and < 4)

	IC2 LOM	IC1 GOM	pUPD11	Unknown
Clinical diagnosis (*n* = 21)	7 (33.3%)	5 (23.8%)	1 (4.8%)	8 (38.1%)
Suspected (*n* = 10)	3 (30.0%)	0 (0%)	0 (0%)	7 (70.0%)
Total (*n* = 31)	10 (32.2%)	5 (16.1%)	1 (3.2%)	15 (48.4%)

*BWS* Beckwith-Wiedemann syndrome, *IC* imprinting center; LOM, loss of methylation, *GOM* gain of methylation, *pUPD* paternal uniparental isodisomy

**Table 3:** The correlation between clinical features and IC2 LOM, IC1 GOM, and pUPD11 defects of the study cohort

	IC2 LOM (*n* = 10)	IC1 GOM (*n* = 5)	pUPD11 (*n* = 1)	*p*-Value
Cardinal features				
Macroglossia	10/10 (100.0%)	4/5 (80.0%)	1/1 (100%)	NS
Lateralized overgrowth	1/10 (10.0%)	3/5 (60.0%)	0/1 (0%)	< 0.05
Exomphalos	1/10 (10.0%)	0/5 (0%)	0/1 (0%)	NS
Hyperinsulinism	0/10 (0%)	1/5 (20.0%)	0/1 (0%)	NS
Wilms tumor or nephroblastomatosis^a^	0/10 (0%)	0/1 (0%)	0/1 (0%)	NS
Suggestive features				
Umbilical hernia and/or diastasis recti	8/10 (80.0%)	4/5 (80.0%)	1/1 (100%)	NS
Ear creases and/or pits	5/10 (50.0%)	2/5 (40.0%)	0/1 (0%)	NS
Nephromegaly and/or hepatomegaly	3/10 (30.0%)	3/5 (60.0%)	0/1 (0%)	NS
Facial naevus simplex	5/10 (50.0%)	2/5 (40.0%)	0/1 (0%)	NS
Birthweight > 2 SDS above the mean	3/10 (30.0%)	3/5 (60.0%)	1/1 (100%)	NS
Transient hypoglycaemia	2/10 (20.0%)	2/5 (40.0%)	0/1 (0%)	NS
Polyhydramnios	1/10 (10.0%)	2/5 (40.0%)	0/1 (0%)	NS
Typical BWS tumors^b^	0 /10 (0%)	0/5 (0%)	0/1 (0%)	NS

^a^ Multifocal and/or bilateral Wilms tumor or nephroblastomatosis

^b^ Neuroblastoma, rhabdomyosarcoma, unilateral Wilms tumour, hepatoblastoma, adrenocortical carcinoma or phaeochromocytoma

*BWS* Beckwith-Wiedemann syndrome, *LOM* loss of methylation, *GOM* gain of methylation, *pUPD* paternal uniparental isodisomy, *NS* no significance

## Discussion

Clinical features and molecular etiology in European and North American BWS patients have been well-studied in the literature. Although the typical clinical manifestations of BWS are macroglossia, macrosomia, abdominal wall defects, and an increased risk of embryonal tumors, a growing body of evidence indicates that not all BWS patients display all of these phenotypic features [1]. Increasing BWS patients in the absence of cardinal features were confirmed by the identification of molecular defects in the 11p15.5 region [12, 13]. Thus, both clinical features and molecular testing are important for the clinical diagnosis and management of BWS.

In the current study, we conducted a single-center retrospective cohort study to characterize the clinical features and molecular defects of clinical suspicion BWS patients in a tertiary children’s care center in Shanghai, China. We showed that the most common cardinal features and suggestive features in clinically diagnosed BWS patients in our cohort were macroglossia (71.4%) and lateralized overgrowth (33.3%), umbilical hernia and/or diastasis recti (65.0%) and ear creases or pits (61.9%), respectively, which were comparable with two previous studies of BWS in the Chinese population [14, 15]. A study [14] of 47 Chinese patients with clinical suspicion of BWS, conducted in Taiwan, showed that the most common major clinical features of clinically diagnosed patients were abdominal wall defects, macroglossia and pre- or postnatal overgrowth, and the most common minor features were ear creases or pits and facial nevus flammeus [14]. A retrospective tertiary-wide study [15] performed in Hong Kong with 27 molecularly confirmed BWS reported that the most common clinical features were macrosomia and macroglossia (70.4%) and abdominal wall defects (70.4%). Since BWS patients present with a wide range of clinical features, a recent study investigated whether clinical presentation varied across BWS patients of different race/ethnicity populations [9]. It was shown that the incidences of macroglossia and exomphalos were higher in BWS patients of European/North American populations than Asian populations, while the incidences of umbilical hernia, organomegaly, and lateralized overgrowth were lower in European/North American populations than Asian populations [9]. The incidences of major clinical features of clinically diagnosed BWS patients in our studied cohort were comparable with previously reported Asian BWS patients [9]. Although an increased risk of developing embryonal tumors during early childhood was reported in patients with BWS [1, 5], BWS-related embryonal tumors were not observed in this cohort. In addition, ART was reported as a risk factor for BWS [16], and one female patient with a BWS score of 6 was conceived by ART in our study.

To date, only a small number of genetically confirmed BWS cases have been reported from mainland, China. In 2013, we reported the first two epigenetically confirmed cases with BWS in Shanghai, China: a female patient with IC2 LOM and a male patient with IC1 GOM [17]. Wang Q. et al. reported two Chinese cases with BWS caused by de novo paternal origin duplication of chromosome 11p15.5 in Shenzhen, China, including one patient diagnosed by prenatal analysis on cord blood [18]. It was shown that IC2 LOM (50–60%) and IC1 GOM (5–10%) in the chromosome 11p15 region occurs in the majority of BWS patients with a known molecular defect [5]. The MS-MLPA test was performed to detect the methylation status of the IC2 and IC1 genes in the chromosome 11p15 region in all patients with clinical suspicion of BWS in this study. Seven clinically diagnosed cases and 3 suspected BWS cases were identified with IC2 LOM, 5 clinically diagnosed BWS children were identified with IC1 GOM, and 1 clinically diagnosed BWS children were identified with pUPD11. A lower incidence of IC2 LOM and a higher incidence of IC1 GOM were observed in our studied cohort than previously reported European/North American and Asian BWS patients [9], which may due to the small size of study cohort. It was shown that 50% of clinically diagnosed BWS patients were identified with IC2 LOM, and 4% with IC1 GOM were identified in Taiwanese BWS patients [14]. Furthermore, molecular studies of Chinese BWS patients in Hong Kong showed that 48.1% of the BWS cases were caused by IC2 LOM, and 11.1% were caused by IC1 GOM [15]. The female patient conceived by ART was identified with IC2 LOM, which was consistent with previous studies [15, 19]. Both CDKN1C mutation and pUPD11 were also observed in previous studies of Chinese BWS patients [14, 15]. Unfortunately, CDKN1C loss of function mutation test was not performed in this cohort at the initial genetic testing, and the parents of the patients refused further genetic testing during the follow-up. Nevertheless, it is important to include CDKN1C mutation test to investigate the molecular etiology of BWS children in our future works.

Several limitations exist in the present study. Firstly, this report describes a single-center retrospective study with a limited number of subjects. Secondly, microsatellite analysis was not performed to further confirm the pUPD11 case identified by MS-MLPA. Thirdly, CDKN1C loss of function mutations test was not performed in this study cohort. It is important to include CDKN1C mutation test and microsatellite analysis to characterize the genotypes of Chinese BWS children in our future works.

## Conclusions

Our study was the first to describe the clinical features and molecular defects of a cohort of 31 clinical suspicion BWS patients in mainland China. Given the large Chinese population and limited reported cases, further studies are needed to investigate the clinical features and genetic mechanisms vary between Chinese population and other well-studied populations.

## Data Availability

The raw data supporting the conclusions of this manuscript will be made available by the authors without undue reservation to any qualified researcher.

## Abbreviations

BWS: Beckwith-Wiedemann syndrome
IC: Imprinting center
MS-MLPA: Methylation-specific-multiplex ligation-dependent probe amplification
IQR: Interquartile range
IGF2: Insulin-like growth factor 2
ART: Assisted reproductive technology
pUPD: paternal uniparental isodisomy
SDS: Standard deviation scores

## References

[1] Brioude F, Kalish JM, Mussa A (2018). Expert consensus document: clinical and molecular diagnosis, screening and management of Beckwith-Wiedemann syndrome: an international consensus statement. Nat Rev Endocrinol.

[2] Beckwith JB (1963). Extreme cytomegaly of the adrenal fetal cortex, omphalocele, hyperplasia of kidneys and pancreas, and Leydig-cell hyperplasia: another syndrome?. Annual Meeting of Western Society of Pediatric Research. Los Angeles, California.

[3] Wiedemann HR (1964). Familial malformation complex with umbilical hernia and Macroglossia--a "new syndrome"?. J Genet Hum.

[4] Mussa A, Russo S, De Crescenzo A (2013). Prevalence of Beckwith-Wiedemann syndrome in north west of Italy. Am J Med Genet A.

[5] Weksberg R, Shuman C, Beckwith JB (2010). Beckwith-Wiedemann syndrome. Eur J Hum Genet.

[6] Choufani S, Shuman C, Weksberg R (2010). Beckwith-Wiedemann syndrome. Am J Med Genet C Semin Med Genet.

[7] Choufani S, Shuman C, Weksberg R (2013). Molecular findings in Beckwith-Wiedemann syndrome. Am J Med Genet C Semin Med Genet.

[8] Eggermann T, Algar E, Lapunzina P, et al. Clinical utility gene card for: Beckwith-Wiedemann Syndrome. Eur J Hum Genet. 2014;22(3):435. 10.1038/ejhg.2013.132 PMC3925261 23820480

[9] Duffy KA, Sajorda BJ, Yu AC (2019). Beckwith-Wiedemann syndrome in diverse populations. Am J Med Genet A.

[10] Priolo M, Sparago A, Mammi C (2008). MS-MLPA is a specific and sensitive technique for detecting all chromosome 11p15.5 imprinting defects of BWS and SRS in a single-tube experiment. Eur J Hum Genet.

[11] Lee BH, Kim GH, Oh TJ (2013). Quantitative analysis of methylation status at 11p15 and 7q21 for the genetic diagnosis of Beckwith-Wiedemann syndrome and silver-Russell syndrome. J Hum Genet.

[12] Gaston V, Le Bouc Y, Soupre V (2001). Analysis of the methylation status of the KCNQ1OT and H19 genes in leukocyte DNA for the diagnosis and prognosis of Beckwith-Wiedemann syndrome. Eur J Hum Genet.

[13] Ibrahim A, Kirby G, Hardy C (2014). Methylation analysis and diagnostics of Beckwith-Wiedemann syndrome in 1,000 subjects. Clin Epigenetics.

[14] Lin HY, Chuang CK, Tu RY (2016). Epigenotype, genotype, and phenotype analysis of patients in Taiwan with Beckwith-Wiedemann syndrome. Mol Genet Metab.

[15] Luk HM (2017). Clinical and molecular characterization of Beckwith-Wiedemann syndrome in a Chinese population. J Pediatr Endocrinol Metab.

[16] Maher ER, Brueton LA, Bowdin SC (2003). Beckwith-Wiedemann syndrome and assisted reproduction technology (ART). J Med Genet.

[17] Zhang T, Xie X, Xu D (2013). Beckwith-Wiedemann syndrome: first epigenetic confirmed case report in China. Clin Genet.

[18] Wang Q, Geng Q, Zhou Q (2017). De novo paternal origin duplication of chromosome 11p15.5: report of two Chinese cases with Beckwith-Wiedemann syndrome. Mol Cytogenet.

[19] Gicquel C, Gaston V, Mandelbaum J (2003). In vitro fertilization may increase the risk of Beckwith-Wiedemann syndrome related to the abnormal imprinting of the KCN1OT gene. Am J Hum Genet.

